# Regulation to Create Environments Conducive to Physical Activity: Understanding the Barriers and Facilitators at the Australian State Government Level

**DOI:** 10.1371/journal.pone.0042831

**Published:** 2012-09-27

**Authors:** Jane Shill, Helen Mavoa, Brad Crammond, Bebe Loff, Anna Peeters, Mark Lawrence, Steven Allender, Gary Sacks, Boyd A. Swinburn

**Affiliations:** 1 World Health Organisation Collaborating Centre for Obesity Prevention, Deakin University, Melbourne, Victoria, Australia; 2 Department of Epidemiology and Preventive Medicine, Monash University, Melbourne, Victoria, Australia; 3 Department of Public Health, University of Oxford, Oxford, United Kingdom; Pennington Biomedical Research Center, United States of America

## Abstract

**Introduction:**

Policy and regulatory interventions aimed at creating environments more conducive to physical activity (PA) are an important component of strategies to improve population levels of PA. However, many potentially effective policies are not being broadly implemented. This study sought to identify potential policy/regulatory interventions targeting PA environments, and barriers/facilitators to their implementation at the Australian state/territory government level.

**Methods:**

In-depth interviews were conducted with senior representatives from state/territory governments, statutory authorities and non-government organisations (n = 40) to examine participants': 1) suggestions for regulatory interventions to create environments more conducive to PA; 2) support for preselected regulatory interventions derived from a literature review. Thematic and constant comparative analyses were conducted.

**Results:**

Policy interventions most commonly suggested by participants fell into two areas: 1) urban planning and provision of infrastructure to promote active travel; 2) discouraging the use of private motorised vehicles. Of the eleven preselected interventions presented to participants, interventions relating to walkability/cycling and PA facilities received greatest support. Interventions involving subsidisation (of public transport, PA-equipment) and the provision of more public transport infrastructure received least support. These were perceived as not economically viable or unlikely to increase PA levels. Dominant barriers were: the powerful ‘road lobby’, weaknesses in the planning system and the cost of potential interventions. Facilitators were: the provision of evidence, collaboration across sectors, and synergies with climate change/environment agendas.

**Conclusion:**

This study points to how difficult it will be to achieve policy change when there is a powerful ‘road lobby’ and government investment prioritises road infrastructure over PA-promoting infrastructure. It highlights the pivotal role of the planning and transport sectors in implementing PA-promoting policy, however suggests the need for clearer guidelines and responsibilities for state and local government levels in these areas. Health outcomes need to be given more direct consideration and greater priority within non-health sectors.

## Introduction

Insufficient physical activity (PA) is the fourth leading risk for mortality worldwide [1] and a major risk factor for overweight and obesity, type 2 diabetes, coronary heart disease, certain cancers and mental ill-health [2]. Despite this, 70% of Australians aged 15 years and over in 2004/05 were classified as either being ‘sedentary’ or having ‘low exercise levels’ (considering exercise frequency, intensity and duration) [3]. Recent research has demonstrated an association between PA levels and environmental factors such as urban density, land use and access to public transport and recreational facilities, including bicycle and foot paths [4], [5]. Policy interventions are essential in addressing inadequate PA levels, as strategies such as education and treatment programs alone are unlikely to result in significant sustained changes in PA [6], [7]. Consistent with a socio-ecological model for health promotion [8], multi-level and multi-sector approaches addressing urban planning, transportation, sports/recreation and settings such as schools and workplaces are required to address obesogenic environments and inadequate PA levels [9].

Understanding the policy setting is essential for the development of coherent and feasible regulatory approaches for creating environments more conducive to PA. The policy literature provides a number of theories and frameworks for explaining and predicting policy processes and outcomes. Two policy science factors were considered to be of particular relevance to this study: governance systems and the influence of stakeholders in the policy-making process. Australia has a federal governance system, comprising a federal parliament, eight state/territory parliaments and over 500 local councils [10]. This structure provides the basis for managing policy and program implementation [11]. The varying influence of stakeholders (both individually and together as networks) and the interactions between competing interests have a major influence on the policy process and outcomes [12]. For example, business interest groups typically have greater influence over government decision-making than other stakeholders due to their contribution to the economy [13].

There has been little empirical research analysing the perceptions of those involved in government policy-making in relation to: 1) promising policy interventions for creating environments more conducive to PA, or 2) barriers and facilitators to regulatory change in this area. Greater understanding and clarity of these factors is an important step in identifying which regulatory options are more likely to be enacted. This paper is part of a wider study investigating policy relating to food and physical activity environments, as well as overarching approaches. We have previously investigated this at the local government level in Australia [14], [15] and at the state/territory level in relation to food environments [16]. The current paper examines findings relating to the PA-policy environment at the Australian state/territory government level. The paper is informed by the following research questions:

1. What do those involved in policy-making at the Australian state government level consider to be promising regulatory interventions for creating environments more conducive to PA?
2. Which of the proposed interventions are most or least supported?
3. What are the perceived barriers and facilitators to implementing the interventions?

## Methods

### Recruitment and participants

A purposive sampling technique was used to ensure reach across: 1) the diverse range of sectors that influence the nutrition/PA environments either directly or indirectly, and 2) all Australian states/territories. Participants were selected by the research team on the basis of having expertise in the development of government policy or expertise relating to food/nutrition or PA. Potential participants were identified from several sources: 1) the study investigators who had considerable experience in the Australian policy arena; 2) high-level officers from government and non-government organizations (NGOs); 3) study participants, who were asked to identify peers in other sectors/states; and finally 4) senior government officers who were asked to nominate a suitable participant within their sector. In total, 45 interviews were conducted with 47 senior representatives (all non-elected bureaucrats, for example, directors, managers or senior policy officers) from government departments, relevant NGOs and statutory authorities (see [16]). Participants were judged by the investigators or the referring people to have extensive policy knowledge related to PA and/or nutrition. A written invitation was followed by a telephone call; with an 84% response rate. For the purposes of this paper, five interviews were excluded from the analysis, as PA was not addressed. In total, data from 40 interviews with 42 participants were included in the analysis. The 42 participants were from nine policy areas (see Table 1). Ethics approval was granted by Deakin (EC 232–2007) and Monash (2007–00–2150) Universities. Participants provided written informed consent prior to participation.

**Table 1:** Summary of interviews by sector and by state/territory jurisdiction.

Area	Number of interviews	State/Territory	Number of interviews
Health/Human Services	10	Victoria	18
Transport	5	South Australia	5
Generalists/NGOs	5	Western Australia	4
Planning	4	Northern Territory	4
Premier and Cabinet	4	New South Wales	3
Education	3	Queensland	3
Environment	2	Tasmania	2
Sport and Recreation	2	Australian Capital Territory	1
Treasury/Finance	2	*N total*	*40*
Statutory Authorities	2		
Primary Industries/Agriculture	1		
*N total*	*40*		

### Data collection and management

In-depth interviews (mean length 61 minutes) were conducted between April 2009 and June 2010. All interviews were on a one-on-one basis, excluding three which were with two participants. All interviews, excluding one, were conducted by the first author (JS). Interviews were conducted in-person for participants in Victoria and via telephone for inter-state participants. Participants did not have access to the questions prior to the interview. All interviews were digitally recorded excluding one where the participant did not consent to recording. Interviews were transcribed verbatim and managed in N-Vivo 8 (QSR International, Melbourne). The interviews comprised three components:

1. A semi-structured component to elicit participants' suggestions for regulatory interventions to create environments conducive to PA;
2. A structured component to identify participants' support for eleven preselected interventions relating to walkability/cycling, public transport, facilities for PA and opportunities for PA (see Table 2). In order to maximise the opportunity for discussion that was not limited to the preselected interventions, participants were questioned about their ideas prior to being presented with the list of preselected interventions. These eleven interventions were based on an extensive literature review of interventions to create environments more conducive to PA and prevent obesity. Interventions were included if they could be reflected in regulatory form and if they were within state government powers. To ensure informed discussion, participants were only questioned about interventions applicable to their department or area of professional expertise (e.g., planning department representatives commented on interventions relating to urban planning).

**Table 2:** Participants' responses to the preselected interventions.

Supported interventions	Caveats
**The interventions most commonly supported in principle were:**	
1. Give active forms of transport more of a priority in the transport system, with less focus on motor vehicles (100% support)	
* That's about making sure that all modes are equal in a way so that pedestrians* *and cyclists aren't disadvantaged, so they have equal priority with cars…So that pedestrians and cyclists aren't viewed as the lower common denominator.* *Everyone has got an equal role and the right to share the road.* (Planning department)	
2. Mandating that school physical activity facilities be made available to the public outside of school hours (88% support in principle)	Not mandated; Feasibility: issues with maintenance, public liability insurance, security.
* Some schools don't want to because there is vandalism, but I think it's about* *again being inclusive and collaborative and working with the school community.* *Because there are some tremendous facilities out there, like tennis courts and basketball courts and fields or ovals that just are not being utilised.* (Education department)	
3. Introducing a congestion levy in the CBD (100% support)	Not applicable to smaller cities, e.g., Darwin, Hobart;
* I think is something we will come to at some point…Look I guess the major* *difficulty is that there is sufficient alternative…our capacity to do more of that is actually constrained by the capacity of the [public transport] network.* (Transport department)	Need to provide an adequate alternative, e.g., improve capacity of public transport
* Yes, that's a good one. In fact there may be a number of very high use roads with some congestion levy, and that's not just to increase physical activity, I mean there would be more around use of public transport, but what it means is people at least walk to and from the bus. And maybe even think about getting on a bike.* (Treasury department)	
**Interventions which were supported but to the lesser degree:**	
4. Enforcing existing physical education requirements in schools (50% support)	Feasibility: completing for time with other curriculum requirements (English, maths, science)
* I certainly think it's something that there has to be a much, much greater push going than there currently is. I think it's laissez-faire…it's not treated as a priority…There's a national curriculum being redeveloped and English,* *mathematics, science and social education are the top priorities. Physical education still hasn't…[been prioritised]. Until subjects like physical education are given the* *same priority status as other subjects, then you're going to be fighting a losing battle.* (Education department)	
**Supported in principle however operationalised differently:**	
5. Restricting parking in the CBD (80% support in principle)	Need to provide an adequate alternative, e.g., improve capacity of public transport;
* What would be far more effective is just to have a proper carbon pricing for parking… Whilst you can say restrict parking, yes…but to what level? How much* *is reasonable?…Whereas a pricing mechanism that said ‘well yes, you can park in the city if you need to park in* *the city, but it's going to cost you to do that'...*(Planning department)	Alternative would be to increase the price of parking rather than reduce the availability.
6. Mandating that a certain percentage of CBD and all major arterial suburban roads have bicycle lanes (56% support in principle)	Need to ensure off-road bicycle lanes as well to target all bicycle-user groups;
* Yes. I would also say with that, we've got to make sure that we have the combination of on road lanes as well as off road lanes. So that we encourage all* *types of users.* (Transport department)	Need to ensure the network of cycling lanes is optimal instead of mandating that a certain percentage of roads have bicycle lanes.
* I don't think it's about a percentage I think it's about getting the network* *right.* (Transport department)	
7. Implement healthy workplace policies for the workplace environment, for example, mandate that employees be provided with time and opportunities for physical activity during work hours (64% support in principle)	Not feasible and needs to be part of broader workplace health policy; Workplaces were however seen as a promising setting for obesity interventions.
* You can't force employees to, ‘okay everyone get up and stretch’, but if you* *have that…softer type of approach of offering employees subsided services that can* *then; you end up with more productive and happier employees as well. Part of thi* *also comes to work/life balance policies and how well they're implemented in the workplace as well…I would have implementation concerns of how you would dothis.* (Premier and Cabinet)	
**Least supported interventions:**	
8. Mandating minimum public transport infrastructure requirements to reduce public transport ‘deserts’ (82% unsupported)	Not viable economically; Need to build on existing public transport networks.
* We're focusing on the urban areas or the areas that we can reliably service, we can't service all areas…So you're never going to be able to effectively service those areas, you have to look at other transport options as well, whether it's ride sharing or car pooling or something like that. We'll never be able to roll out public transport everywhere…also we find that in cases where we have previously they're not widely patronised in any way. So it's not just about cost, it's about what is the need and particularly in rural areas, the public transport can't really meet people's needs in a way.* (Planning department)	
9. Subsidising the cost of public transport to the consumer (80% unsupported)	
* …our preference is to actually increase frequency rather than lowering the cost.* (Planning department representative)	
* …it is fairly heavily subsidised already...I can't see [the Transport Department] coming at that one very happily.* (Transport Department)	
10. Set standards for the required levels of both indoor and outdoor play equipment for different sized schools (83% unsupported)	Must also provide funding to implement this initiative.
* So what's going to actually make the difference?…I don't think it's [about] having three swimming pools or ten basketball courts or whatever. It's actually having the drivers on the ground who are passionate about an area, and policy in place, and being able to say, “This is what we're told to do, this is what we will do”…Having said that, there are schools out there without facilities at all, so maybe we need to look at a starting point.* (Education Department)	
11. Subsidising the price of commuter bicycles (and associated essential bicycle equipment) (70% unsupported)	
* I'm not sure it's the bikes that need subsidy…what are the barriers to people riding bikes?…it's not price, it's [that the] roads aren't safe, and when I get to work is there a shower? Is there somewhere to park?* (Treasury Department)

1. A semi-structured component was again used to identify participants' perceptions of the barriers, facilitators and feasibility relating to the interventions discussed.

In this project, regulatory intervention was defined as any intervention by government that was either legislated or enforced through some other means, for example, conditions attached to funding.

### Analysis

Data were analysed by two researchers (HM, JS) using constant comparative [17] and thematic [18] analyses. Collaborative analysis was employed with the two researchers analysing themes independently and then together to reach consensus. Analysis was conducted at three levels: First, an analysis was undertaken incorporating participants': 1) suggestions for regulatory interventions and 2) responses to the eleven preselected interventions. Second, transcripts were analysed to determine themes that cut across both of these areas. Finally, findings were related to relevant theory and knowledge.

## Results

### Regulatory interventions suggested by participants

The regulatory interventions that participants suggested to create environments more conducive to PA could be categorized into two main areas: 1) urban planning and provision of infrastructure to promote active travel; and 2) discouraging the use of private motorised vehicles. Specific interventions suggested under these two areas are presented below. Only one of the interventions frequently suggested by participants to create environments more conducive to PA, also appeared on the list of interventions preselected by the research team. This was: prioritising active transport over motorised vehicles. Participants frequently suggested a further three interventions: provision of facilities to promote walking/cycling, provision of safe useable open spaces for recreation/active transport and changing salary packaging arrangements which currently encourage private vehicle use.

#### 1. Urban planning and provision of infrastructure to promote active-travel

Over half of participants suggested improved urban planning and provision of PA-promoting infrastructure as potential solutions for creating environments more conducive to PA. A quarter of participants suggested it should be mandatory for all new developments to provide facilities to promote walking/cycling. This comment made by a Transport department representative was typical:

*I think for new commercial buildings to have end-of-trip facilities, so that's your bicycle storage and showers and changing facilities and lockers, etc, in new buildings. We've been looking at trying to require all the new government buildings and lease buildings* [to] *have those facilities as well, and providing some design guidelines to the planning scheme as to what they should look like…*

Over one third of participants suggested planning regulations to ensure adequate provision of safe, useable open spaces and foot/bicycle paths in order to encourage active transport. The following comment was made by a Transport department representative:

*…it is about the things that will encourage active transport, so look I think I would probably focus on the facilities for active transport which is largely about facilities for cycling; facilities for walking are straightforward. But the other thing that makes a difference here is…the nature of the urban form. Now this is not a quick fix and anything that requires you to change the urban form, well sit back and work really hard on it and in 30 years time you'll start to see some benefit but we have to do it…*

Participants from a number of sectors (specific area of responsibility or service provision, for example, health, planning, transport) commented that retrofitting existing buildings or developments was not feasible due to the costs involved.

Several participants suggested that housing growth should occur near existing public transport, decreasing the reliance on private vehicles. One of the preselected interventions, ‘mandating minimum public transport infrastructure to reduce public transport deserts’, was generally viewed as being economically unfeasible. Instead, the strategic location of new housing was suggested as an alternative. The following quote from a Planning department representative was typical:

*So the first thing is putting a lot more housing near public transport, changing the planning rules to allow for multi storey developments along traffic corridors where there is public transport…Of course when you increase the number of people…using public transport, then the frequency of the service can increase as well because there'll be a greater patronage to support that.*

#### 2. Discouraging use of private motorised vehicles

The second dominant category of interventions suggested by participants was discouraging the use of private motorised vehicles by prioritising active forms of transport over motorised vehicle use. The following quote from a Planning department representative was typical:

*I would look at some market-based instruments that discourage private vehicle usage. Seems to me it's all too easy just to jump in the car and drive 100 metres to the supermarket when you could've walked. I would be intrigued to see what would happen…if we actually had the courage to pedestrianise more of the city and make it more difficult for vehicles to dominate the road space. I'd love to see a more conscious investment programme in public transport options. My sense is the balance is still very much in favour of road/private vehicle* [use].

While participants commonly spoke about the need to discourage the use of private motorised vehicles, only one specific intervention was frequently suggested. This was changing the salary packaging/fringe benefit arrangements which currently encourage private vehicle use. A quarter of participants suggested that changing the arrangement would encourage more active forms of transport, as this NGO representative explained:

*One of the ones that has been kicked around for a while and I think has enormous potential, and hasn't been taken advantage of, is things like the change in the tax structure, which currently encourages employers to give mid-ranking and senior staff motor vehicles as part of their salary packages. A gold public transport pass for example – those are the kinds of things that are very ‘do-able’ within a tax structure, to provide that incentive* [to use more active forms of transport].

The other interventions that were suggested by one or two participants to discourage the use of private motorised vehicles were: introducing congestion zones, reducing or increasing the cost of parking and 'pedestrianising' roads/areas. The following comment was typical:

*You'd reduce your car parking. Or you do the inner city tax on – I think London does it – where you've got to pay more to be driving in the inner city…* (Transport department).

### Participants' responses to the eleven preselected interventions

Overall, four of the eleven preselected interventions (provided by the research team) were supported (participant agreed in principle, that the intervention could potentially be an effective or useful means of creating environments more conducive to PA) by over 50% of participants, and in the majority of cases by over 80% of participants (Table 2). Interventions promoting walkability/cycling and facilities for PA received support from the most participants. These were: prioritising active forms of transport in the transport system; making school facilities available to the public outside school hours; introducing a congestion levy in central business districts (CBDs); and to a lesser degree, enforcing existing physical education requirements in schools. While ‘support’ was often provided, there were frequent caveats (Table 2). For example, while participants supported ‘making school facilities available to the public outside of school hours’ they did not support it being mandated. Further, participants supported the use of school facilities provided that agreements were in place regarding maintenance, public liability insurance and security. Participants perceived that some interventions were less applicable in certain jurisdictions, for example, introducing a congestion levy in the CBD was less applicable to smaller Australia cities (e.g., Hobart, Darwin).

Three interventions were supported in principle, that is, participants supported the aim of the intervention; however, they suggested alternate ways to achieve a similar outcome. For example, restricting parking in the CBD was supported in principle, however participants suggested increasing the price of parking to discourage use as another means of achieving a similar outcome (see quotes in Table 2).

The remaining four interventions were least supported. Less than half of participants supported: subsidising the cost of public transport; subsidising the cost of bicycle-related equipment; mandating minimal public transport infrastructure to reduce public transport ‘deserts’; and setting standards for the required levels of both indoor and outdoor play equipment for different sized schools (see Table 2). These interventions were either perceived as not economically viable or likely to be ineffective in increasing PA levels.

### Barriers and facilitators to PA-promoting policy change

Six themes emerged around barriers and facilitators to achieving regulatory change in relation to creating environments more conducive to PA.

#### Relative power of the road lobby

Participants mentioned the ‘road lobby’ as a major barrier to introducing regulations to prioritise active transport over motorised vehicle use. The constituents of the ‘road lobby’ were not clearly defined; however mention was made of the car industry, road users/tax payers and automotive associations. A Transport department representative spoke about the influence of the road lobby on government decision-making when discussing reducing vehicle speed limits to encourage walking/cycling:

*Politically it* [reducing speed limits] *would be a nightmare. The car, road lobby is still quite strong. I'd say a lot of local councils are still probably anti that. So…your…inner city local government[s], I reckon would be supportive completely – they've got more walkers and cyclists and they're sick of having lots of traffic through their streets. But I think your outer suburbs would hate to hear that we've got to drop down to 30 or 40 *[km/hour]. *So politically difficult…*

#### The planning system

Participants discussed the role of planning in creating environments more conducive to PA and the need for the planning system to be strengthened. This quote from a Planning department representative was typical:

*Probably the planning system as well is not as tight as it could be…so for example, you've got development occurring in areas which aren't very accessible. So out of urban areas where there isn't public transport or you can't walk to school… So* [there's] *really a lack of integration between infrastructure,* [the] *planning system and the land use planning system.*

There was also discussion about planning systems not adequately considering public health outcomes, as a Transport department representative commented:

*There's a great lack of understanding of how transport and land use is integrated. There is generally no understanding of health and planning.*

Participants generally acknowledged the potential of the planning system to consider health outcomes, however it was suggested that the system was constrained by planning guidelines being open to individual interpretation. For example, when discussing a clause within a planning scheme that related to neighbourhood ‘walkability’, a NGO representative commented:

*…we've got* XX [a number of] *councils, we've probably got* XX [a number of] *different ways that's being interpreted- from some people probably applying it pretty rigorously to other people being pretty open-ended* [in] *the way they apply it. So you're…getting the picture that the system in one sense has got a lot of the capabilities to deliver what we're talking about* [public health], *but a lot of it depends upon the will of the individuals and players within the system.*

Part of the problem appeared to be due to a lack of clear definition of ‘walkability’. This comment from a transport department representative was typical:

*…planning schemes by and large require a certain amount of walkability; walkability is one of those things that is obviously hard to define. We're doing some work at the moment to try and give councils, planning authorities some guidance about that.*

When discussing the possibility of requiring that ‘brownfield’ project developers (urban renewal projects) contribute towards infrastructure to promote walking, the same participant highlighted issues at the local government level:

*…local government tends to seek developer contributions for various things; sometimes it's transport…In some places they do it well and in other places they do it badly, so it's certainly possible. Can we do it uniformly? That's probably a little bit* [unfeasible]*…but it should be the goal… Council's willingness to go toe to toe with developers* [determines how successful seeking developer contributions is]*…there are some places that need the economic stimulus of a particular development so, you know,* [Council will say to developers,] *‘do whatever you want’.*

Some participants suggested solutions to facilitate the uptake of health agendas in different professions and government areas, for example, this Planning department representative suggested the following:

*So sharing knowledge I guess, and a capacity building mechanism....And I think that looking at all layers of opportunities, so for instance…lecturing to urban planning students... about the relationship between health and the built environment, and…talking about what things planners can do to create healthier communities…It's* [building] *capacity, but it also tells people who are coming into the profession that ‘this is the kind of thing that we do. This isn't somebody else's job, this is actually part of your job, part of what we take responsibility for’.*

#### Cost

Participants suggested that the potential cost of implementing regulations was a barrier to achieving change, particularly with regards to urban planning and public transport. For example, ‘mandating minimum public transport infrastructure requirements to reduce public transport ‘deserts’’ was generally not supported, as it was perceived as being economically unviable (see quote in Table 2). Furthermore, cost was also seen as a barrier to the establishment of bicycle lanes, as discussed by a Planning department representative:

*The main barriers* [to the establishment of bicycle lanes] *usually are cost, in the sense that you're retrospectively taking back road space or other space. That's incredibly expensive…*

#### Evidence

The availability of evidence to support the effectiveness of policy interventions in making the environment more conducive to PA was seen as a facilitator to change by many participants. A NGO representative spoke about the importance of having evidence when discussing factors that could facilitate departments to re-orientate priorities, for example, Departments of Transport or Planning prioritising walking, cycling and public transport over roads and motorised vehicles:

*…we need to have the quality research and evidence at our disposal, evidence of effectiveness so that when you're arguing for these things you have that evidence at your disposal. I think we need to mobilise our advocacy efforts better and be putting up consistent and well articulated and well supported arguments.*

A Transport department representative highlighted the imperative of economic evidence, suggesting the need to generate evidence around the value of public transport versus road development in terms of the potential health benefits:

*… transport projects largely get their economic benefits from savings and time…so we do these business cases and we do benefit cost ratios...One of the things we've never done before but we'd like to do, is to have a look at some of the health benefits associated particularly with public transport projects, which are every public transport trip starts with a walk…if we understand the value of the amount of additional walking that that creates and we understand the health link between that additional activity and health and what the economic benefits are to society, that gives us a sense of ‘well if we build a train line we get this slither of health benefit as well whereas if we build a freeway we don't get any of that’.*

When discussing potential interventions for making environments more conducive to PA, a Treasury department representative spoke about the challenge of implementing policy interventions when evidence relating to impact was inadequate:

*…the other thing that comes* [into consideration with]*…food systems, physical activity…is* [that] *the long term impact of short term actions are not very clear.*

#### Collaboration across sectors

Participants also indicated that collaboration between sectors was an important facilitator to change in relation to policy to support environments for PA. A Planning department representative discussed the lack of collaboration within government:

*I think one of the things that hasn't necessarily happened so well in the past is…having planning departments and transport departments working together. It seems like a logical thing but it doesn't necessarily always happen…they all operate as silos, they're not necessarily making good decisions in conjunction with each other. And if you're talking about creating an environment that supports more physical activity, then you've got to look really hard at your public transport system* [and urban planning] *and try and make the two work together.*

#### Synergies with climate change/environment agendas

A number of participants discussed the synergies between policy aiming to increase PA levels and policy aimed at addressing climate change and environmental issues. Several participants suggested that the uptake of policies was more likely if they delivered on more than one outcome, namely climate change or traffic congestion. The following comment was made by a Premier and Cabinet department representative when asked what they considered as the most promising intervention to make environments more conducive to PA:

*I think I lean towards the infrastructure changes in relation to active transport* [as a promising intervention]. *I guess there's multiple reasons: 1) it can have an impact across a whole range of different audiences. So it's not specific to a particular target group* [and] *2) it ticks a few different boxes. So it ticks the climate change box, it ticks the preventive health box…the workplace productivity etc etc, so there's multiple wins there…If what you are doing, or what you are proposing can be sold to different decision-makers based on evidence around their particular area of influence…then I think there's multiple benefits there.*

Furthermore, a NGO representative discussed the importance of collaborating with the environment sector to strengthen advocacy for the uptake of policies to make environments more conducive to PA:

*And one of the things I think we haven't done very well is build a common cause with…the environment movement. Because there are so many options on a policy front that are a benefit in terms of obesity reduction which are equally important in terms of climate change and we haven't taken best advantage of those. We don't team up with our climate change friends to support public transport initiatives…to support cycling and walking...Those active transport options have a range of community benefits. And we keep battling away in our little obesity world and they keep in their somewhat bigger and probably more influential climate change world. There seems to me to be opportunities for us to work together.*

## Discussion

This study aimed to identify potential regulatory interventions for creating environments more conducive to PA, and barriers/facilitators to their implementation at the state/territory government level in Australia. The regulatory interventions frequently suggested by participants fell into two main areas: urban planning and provision of infrastructure to promote active-travel, and discouraging the use of private motorised vehicles. In response to the set of preselected interventions for which the states/territories had authority, interventions promoting active transport over private vehicle use and facilities for PA generally received the strongest support, for example, mandating that school PA facilities are available to the public outside of school hours. The least supported interventions included subsiding cost of public transport, subsiding cost of bicycle-related equipment and mandating minimal public transport infrastructure to reduce public transport ‘deserts’. These were perceived by participants as not economically viable or unlikely to be effective in increasing PA levels.

Only one of the interventions frequently suggested by participants to create healthier PA-environments, also appeared on the list of interventions preselected by the research team. This was: prioritising active transport over motorised vehicles. This agreement between participants' ideas and a preselected intervention suggests that the ‘prioritisation of active transport over motorised vehicles’ is an especially promising intervention. The lack of concordance between participants and the research team on other interventions may reflect the heterogeneity of the states/territories of Australia in terms of policy need, or alternatively, suggests that there is no clear agreement on the best policy options to address inadequate PA levels. The main barriers to implementing policy to create healthier PA environments were: the power of the ‘road lobby’, weaknesses in the planning system and the cost of potential interventions; whereas the need for evidence, collaboration across sectors and synergies with climate change/environmental agendas emerged as facilitators. Douglas et al [19] suggest that rising car use reflects and reinforces the physical and social environments that have been created. They suggest that cars are the “new tobacco” and note similarities in the tobacco and car lobbies in terms of car/tobacco use being seen as an individual and aspirational choice. Douglas et al. suggest that well-coordinated advocacy in order to build public awareness and engage with policy makers over time is essential to counteract the car lobby and thus increase active transport and reduce car use. Furthermore, policy makers in Canada believed that systemic factors were the main barriers to creating a healthy built environment for youth, specifically economic (governments having mandates but insufficient resources); communities inheriting built environments designed to facilitate car use; governance (distinct and competing mandates); and cultural factors (aspirations and acceptability and safety of active transport) [20].

The results from this present study show some similarities with the findings of a series of studies conducted in nine European countries which analysed stakeholder opinions (including representatives from government, NGOs, food/fitness industry and public health specialists) on policy options for addressing obesity [21]. Participants were asked to compare the performance of several policy options. Cross-national results showed that of the options relating to PA environments, stakeholders scored ‘improving communal sporting facilities’ most highly, whereas ‘changing planning and transport policies’ was less popular [21]. The less popular options were considered costly, with very long timeframes for implementation [21]. Our participants also cited cost as a barrier; however, in contrast to the European study, our participants commonly suggested planning and transport policy initiatives to promote PA. This may reflect different local environmental contexts, for example, European countries typically have higher density cities with greater mixed use developments and more active transportation. The differences in findings highlight the importance of conducting analyses at the local level.

This study highlights the pivotal role of the planning and transport sectors in implementing policy initiatives to create environments more conducive to PA. In general, most of the interventions suggested and supported by participants fell within the jurisdiction of the planning and transport sectors. Participants cited two issues in the planning system as barriers to achieving PA-promoting policy: that planning schemes do not explicitly address health outcomes and that planning schemes are open to interpretation by the local government. In Australia, state and local governments share responsibility for planning schemes between them. Although the exact delineation of powers varies among the states, state governments set overall planning policy and general planning standards, with local governments responsible for more specific policy and the consideration of individual planning applications (see for example the Victorian Planning and Environment Act 1987 [22]). Consequently, many of the interventions suggested and supported by state government participants will depend upon local governments for implementation, making their feasibility dependent upon both levels of government. The sharing of roles makes the creation of well-implemented planning policy difficult, for example, even if the state guiding policy is health supportive (or at least neutral in being neither supportive nor damaging) there is sufficient scope for councils to prefer developments that do not promote health. Equally, there is the capacity for local government to implement policy initiatives to promote healthier PA environments. For example, in Victoria Australia, the Port Phillip City Council's Municipal Strategic Statement states that it will ‘create an integrated and sustainable transport network which supports the use of public transport, cycling and walking above private car travel’ [23]. The devolution of responsibilities for planning in Australia (whereby state and local governments share responsibility for planning schemes, rather than a centralized ‘top-down’ approach), highlights the necessity for a whole-of-government approach in creating environments more conducive to PA. Potential for inconsistent approaches between state and local governments suggests the need for more clearly defined roles and responsibilities.

The creation of a built environment which is more conducive to PA was highly supported by participants. In particular, participants thought that planning controls should be strengthened to encourage development around existing infrastructure (for example, public transport) and to discourage continued urban sprawl via increased housing density. The importance of changes of this type is well-documented in the literature and has been incorporated in some jurisdictions with varying success. In Victoria, for example, the Melbourne 2030 plan (released in 2002) specified certain areas of Melbourne as ‘Activity Centres’ – areas where there was existing infrastructure and development should be encouraged [24]. In Melbourne as elsewhere, these policies are some of the obesity prevention-related policies with the highest level of public resistance. Local communities have strongly resisted medium to high-density developments, despite the available infrastructure. As a result, local governments have been reticent to promote development of the sort recommended by state government policy and supported by this study's participants.

The regulatory picture is further complicated by the government's funding of transport. In 2010–11 the federal government provided $935.3 million in funding for local roads [25]. This funding was complemented by funding for the National Transport Plan which amounts to $36.2 billion over 6 years with $9 billion in 2011–12 [26]. Both road and rail projects are funded under the National Transport Plan, although rail projects are focused in rural and regional areas and not in the areas of urban sprawl identified as ‘public transport deserts’ by participants. Given that potential PA-enabling policy levers, including funding, sit across all three governance tiers, the importance of vertical coordination between tiers of governance is highlighted. In accordance with this, our findings at the local government level found that funding provided from the state and federal tiers of government influenced policy enactment at the local level [14]. In this study, participants at state government level frequently linked PA regulatory actions to the local government level, however there was less frequent discussion of linking up to the federal level. This is in contrast to the food component of this study, whereby state level participants frequently made reference to the federal government but less frequent reference to the local government [16].

Participants believed that collaboration across sectors was required to facilitate PA-promoting policies. The imperative for horizontal coordination across government is clear given that many of the PA-promoting policy levers sit within the jurisdiction of transport and planning. Furthermore, it is clearly in the interest of other sectors to increase population rates of PA, for example, the transport and environmental sectors will benefit from reduced traffic congestion [27] and carbon emissions [28], [29] associated with less private vehicle use, respectively. These sectors could contribute to a whole-of-government policy approach by investing in education and research interventions to promote PA and investigate the health and environmental benefits that would accrue. Many governments recognise the importance of ‘whole-of-government’ or ‘joined-up approaches’ for addressing preventive health issues including obesity [30], [31], however few successful examples exist. A promising model however, is that of the Health-in-All policies approach introduced by the South Australian state government in 2007 [32]. This approach uses governance and accountability structures and a ‘health lens’ analysis process as mechanisms to achieve improved population health outcomes [32]. Early evaluations indicate successful engagement of other sectors in achieving health outcomes [33].

The varying influence of different stakeholders and the interactions between competing interests is widely recognised in policy-making theory and practice [34]–[36]. Participants in our study cited the ‘road lobby’ as a barrier to implementing policy in relation to active transport initiatives and de-incentivising private vehicle use. The constituents of the ‘road lobby’ were not clearly defined by participants. Indeed, beyond government, its associated statutory agencies and state automobile associations, it is hard to identify who this lobby is. It is likely that government itself is its own most significant ‘lobby’ in favour of roads, which are considered essential for national and regional economic growth. For example, in Victoria, Australia, the freight and logistics industry alone contributes 14.7% to Gross State Product [37], with congestion estimated to be costing the economy between $1.3–2.6 billion/year [38]. Given the Australian government's commitment to growing business productivity [39], it could be assumed that protecting and building the roads network is associated with economic productivity. In order to achieve a population shift to more active forms of transport it is likely that the conventional approach to transport planning will need to change, that is, prioritising cycling/walking over motorised vehicles, as is evident in several Northern European cities.

Participants perceived the potential cost of interventions to be a barrier to change in this study, with several interventions being unsupported by participants on the grounds that they were not economically viable, e.g., provision of public transport infrastructure requirements to reduce public transport ‘deserts’ or interventions which required retrospectively changing the composition of the city landscape (e.g., establishing walking paths or areas of open space). Financial considerations were also one of several barriers identified by Canadian policy makers when examining barriers to optimising investments in the built environment [20]. Australian cities have low population densities and urban sprawl, therefore increasing car-dependency and making public transport and infrastructure provision challenging and costly. It is interesting that participants perceived greater public investment in public transport as economically unviable; whereas, the conventional mindset in Australia accepts enormous investment in roads. This suggests that active transport appears to be less of a priority than motorised vehicles and roads. In order to achieve increased population levels of PA, it is likely that a better balance between health and environmental agendas and economic agendas needs to occur. These findings suggest that it will be important for the public health community to engage in more detailed discussions with government, particularly regarding the downstream health costs associated with not improving our PA environment.

One of the difficulties with implementing regulatory interventions to create environments more conducive to PA is that policy options require serious long-term investment both politically and financially. This makes policy uptake challenging. One of the challenges is the impact of policy cycles on long-term planning and investment, where government is unlikely to see the benefits of policy implementation within their time in office. For example, for many of the potential PA-promoting policy solutions, it is unlikely there will be immediate measurable effects, such as those within urban and transport planning which require changes in the city landscape and shifts in social norms. These findings highlight the importance of having greater dialogue between the public health community and government regarding the most optimal way to frame existing and potential evidence for such long-term issues involving environmental change.

In addition to collaboration across sectors, participants in our study spoke about a further two strategies to improve uptake PA-related policy. One solution suggested by participants to facilitate the implementation of PA-promoting policy was maximising opportunities associated with climate change/environmental agendas. Several participants spoke specifically about the synergies between PA-promoting policy and climate mitigation policy, highlighting the importance of recognising synergies or co-benefits in policy-making and using these synergies to leverage support with other sectors. The potential for policy co-benefits provides an additional rationale for policy-makers. PA appears to receive less attention than food in the obesity debate [40], [41]; a notion also discussed by some participants in this study. Given the current political focus on climate mitigation, climate change and environmental policy offers an excellent opportunity for the public health community to collaborate across sectors to strengthen advocacy for the uptake of policies to create environments more conducive to PA.

Another solution offered by participants to facilitate the implementation of PA-promoting policy was the generation of evidence. The generation of evidence is a challenge in relation to policy interventions for creating environments more conducive to PA, as many of the solutions (suggested by our participants and others), such as introducing congestion zones and the provision of showers/lockers in workplaces, have indirect associations with PA, that is, potential effect on PA is mediated via other factors. Showing an association between policies and positive behavior change such as increased PA levels is difficult. Furthermore, evidence is however, only one factor in the complex policy-making process [34], [42]. Evidence is used by government in different ways [43] depending on the ideologies of those who influence policy [44]. As Russell et al. highlight, ‘the selection, evaluation and implementation of research evidence are important in the policy-making process, [but] they do not equate to that process’ [45]. It is also important that evidence-producers make evidence accessible (timely, appropriate language) to policy developers [46].

The strengths of this study include the involvement of those involved directly in the policy-making process, and the breadth of stakeholders interviewed. To date, this project has investigated the local and state government tiers; once the federal government data collection phase is complete, further analysis will be undertaken into how policies and actions between governance tiers could be more closely aligned. One of the limitations of the study was pre-selected interventions presented to participants (prioritising active transport over motor vehicles) lacked specificity and may have been better classified as a policy direction rather than specific approach to create environments more conducive to PA. While this study was undertaken in the Australian context, we believe that some of the findings are more widely transferable as several factors, such as car-dependency, are common in other countries. However, uniquely local characteristics which may affect the feasibility of policy options, such as Australia's low population-density and high urban sprawl, may limit the transferability of our findings. As this research is exploratory in nature, the findings are intended to highlight areas for further analysis, and indicate potential barriers/facilitators for policy change – these will need to be explored in different contexts to assess their relevance.

In order to develop coherent and feasible policy approaches, it is necessary to understand the barriers and facilitators to policy change. This study gives an ‘inside view’ from those involved in the policy-making process at the state/territory level of government in Australia. Similar to our findings in the food component of this study (see [16]), the implementation of many of the interventions suggested by state government participants, are largely the responsibility of the federal government and local governments. This finding is not surprising with respect to urban planning in particular, given the shared responsibility between state and local governments as discussed previously.

### Recommendations

The findings of this study highlight the necessity for a whole-of-government approach to address inadequate PA levels. Planning and transport sectors have a pivotal role in implementing PA-promoting policy. We would recommend that:

1. planning processes are strengthened by establishing clearer guidelines and responsibilities for state and local governments with respect to the promotion of PA;
2. health outcomes are explicitly included in the planning and transport sectors. This could be in the form of a health impact statement and/or incorporating health gains into the cost-benefit analyses undertaken to prioritise transport investments. Benefit assessments which focus on moving people and gaining health rather than reducing travel time and costs which incorporate harms to health and the environment are more likely to favour active transport than existing assessments.
